# Labor market participation among patients referred to occupational medicine with low-back pain: a Danish nationwide register-based cohort study

**DOI:** 10.5271/sjweh.4305

**Published:** 2026-09-01

**Authors:** Mathias Moselund Rønnow, Jesper Medom Vestergaard, Dorte Rytter, Morten Fenger-Grøn, Morten Vejs Willert, Johan Hviid Andersen, Marianne Kyndi

**Affiliations:** 1 Department of Occupational and Environmental Medicine–University Research Clinic, Danish Ramazzini Center, Goedstrup Regional Hospital, Herning, Denmark.; 2 Department of Clinical Medicine, Faculty of Health, Aarhus University, Aarhus, Denmark.; 3 Department of Public Health, Research Unit for Epidemiology, Aarhus University, Aarhus C, Denmark.; 4 Medical Diagnostic Center, University Clinic for Innovative Patient Pathways, Silkeborg, Denmark.

**Keywords:** musculoskeletal disorder, occupational epidemiology, occupational medicine, return to work, propensity score

## Abstract

**Objectives:**

This study aimed to characterize long-term labor market participation from five years before to five years after assessment at departments of occupational medicine among patients referred with low-back pain (LBP) and to compare these patterns with those in a matched general working population. Secondary objectives were to assess subgroup differences and time to return to work.

**Methods:**

In this nationwide register-based cohort study, we included Danish residents aged 18–60 years referred for assessment (N=8256) and matched comparators (1:5 on sex, age, and calendar year; N=41 280). Using weekly register data, we calculated the prevalence of different labor market states before and after assessment as well as propensity score-weighted prevalence differences and ratios, and we performed stratified analyses. We estimated the five-year cumulative incidence of return to work among individuals on temporary public benefits using the Aalen–Johansen estimator.

**Results:**

At assessment, 37% of patients were working versus 83% of comparators. Five years post-assessment, 40% of patients were working and 32% received permanent health-related public benefits. We observed substantial heterogeneity, with larger deficits in work participation in several subgroups. Among patients on temporary public benefits at assessment, the five-year cumulative incidence of return to work was 42%, with no increase in overall work prevalence.

**Conclusions:**

LBP patients had persistently poorer labor market participation than matched comparators, with declines already evident one year before assessment. Prognosis was particularly poor among older patients, those with weaker labor market participation at assessment, and those with comorbidities. These findings highlight the need for early identification and timely intervention and referral.

Low-back pain (LBP) is a major and increasing global health problem (1) and the leading cause of years lived with disability (2). It is a major reason for absenteeism from work, causing a huge health burden and massive economic costs (3–8). While most patients with LBP experience symptom recovery during the first six weeks, improvement tends to slow thereafter (9). Work-related outcomes seem to follow a similar course: approximately 93% of workers return to work within the first siz months, whereas an estimated 7% remain on long-term work absence (10). However, these estimates vary markedly across studies, populations and settings (10), and less favorable work-related outcomes have been observed among populations with more severe or persistent LBP (9, 10). In a study of patients with chronic non-specific LBP, only 52% reported high work participation after one year (11). In a Scandinavian context, a large Swedish register-based study found that patients receiving specialized outpatient care for LBP had 45 excess days of sickness absence and disability pension during one year and 2.6-fold higher productivity losses when compared to general population matches (12).

Several predictors of reduced labor market participation have been identified in LBP patients, including poor general and mental health, lower socioeconomic status, increasing age, disability, higher pain intensity, higher physical work demands, adverse psychosocial work factors, improper pain coping, fear of movement, low recovery expectations, and lifestyle factors, although their relative importance varies with symptom duration and across settings (13–16). The heterogeneity in prognosis and prognostic factors across settings underscores the importance of setting-specific evidence. Yet, evidence from occupational medicine settings remains sparse. In a Dutch study of 5472 workers who consulted an occupational physician for LBP, 82% had not achieved occupational recovery within 30 days, 10% within 1 year, and 2% within 2 years (17). During the 2-year follow-up period, the mean duration of sickness absence was 151 days, and the mean cost per episode reached up to €23 000 depending on LBP subtype (17). Similarly, in a Canadian inception cohort of 305 workers receiving workers’ compensation for an LBP-related injury, 11.3% had not returned to work after 270 days (16).

Although informative, the available studies were limited to follow-up periods of ≤2 years and conducted in settings that are not directly comparable to Scandinavian occupational medicine, where services are often hospital-based and accessible through the public healthcare system. There is therefore a need for studies describing longer-term work participation and prognosis, particularly in hospital-based occupational medicine settings. Evidence from settings not based on employer-linked occupational health arrangements may provide additional insights less influenced by selection related to access and referral. Moreover, although the first month of sickness absence has been identified as a critical window for preventing prolonged work absence (10), little is known about how labor market participation develops before occupational medicine assessment.

Denmark’s publicly funded healthcare system and comprehensive national registers offer a unique opportunity to study these labor market dynamics. In Denmark, all individuals have free and equal access to hospital departments of occupational medicine, to which they may be referred by a general practitioner, medical specialist, or other relevant stakeholders when work-related factors are suspected to contribute to the onset or persistence of disease. The departments of occupational medicine then systematically assess thecase work-relatedness and provide counselling to support sustainable employment. However, counselling in this setting is constrained by the limited evidence on long-term labor market participation prognosis.

To our knowledge, no other studies have evaluated long-term work participation patterns both before and after occupational medicine consultation for suspected work-related LBP or tried to identify vulnerable subgroups in this setting.

Therefore, our primary objectives were to (i) characterize long-term labor market participation from five years before to five years after assessment at departments of occupational medicine among patients assessed for LBP and (ii) compare these patterns with those in a matched general working population. Secondary objectives were to (iii) assess subgroup differences and (iv) analyze time to return to work among patients receiving temporary public benefits at assessment.

## Methods

### Study design and setting

This nationwide register-based cohort study is part of a larger research project, “The Danish Occupational Medicine Cohort”, comprising all patients seen at departments of occupational medicine in Denmark during 2000–2017 (18) and a matched general population reference group. Patients and their matches were followed from five years before to five years after patient assessment at an occupational medicine department, with follow-up spanning 1995–2022.

The Danish departments of occupational medicine are hospital-based specialist departments that assess work-relatedness of diseases and provide counselling to help patients sustain or regain their labor market participation. Access requires referral, usually from a general practitioner or medical specialist and, in some cases, from a labor union.

In Denmark, all residents have access to a free publicly funded healthcare system. A unique and extensive infrastructure of nationwide clinical and administrative registries contains detailed information on all citizens. Information from different registries can be linked through the unique personal civil registration number (CPR in Danish), which is assigned to all citizens in Denmark (19). The CPR is also used to identify citizens who qualify for receipt of public benefits from Danish authorities in cases of unemployment, disability or disease (20). In the Danish context, public benefits refer to public cash benefits paid to individuals when they are not fully self-supporting through ordinary employment, for example during sickness absence, unemployment, parental leave, disability, or retirement. Because Denmark has a tax-financed welfare state with extensive income-replacement schemes and corresponding administrative registers, data on public benefits are widely used as indicators of labor market status.

Reporting of this study followed the STROBE guidelines (21, 22).

### Data sources

For this study, we used information from several nationwide registers.

*The Danish Register for Evaluation of Marginalization (DREAM)* (20) provides high-quality information on all transfer payments and public benefits provided to Danish citizens (20, 23). It contains numerous codes for different public benefits and information on immigration and death, which is updated weekly. No weekly code indicates no public benefits which most likely means income from labor. For sickness absence, only spells registered in the public transfer payment and benefit system are captured; employer-paid sick leave during the initial four weeks is therefore not recorded, and short-term sick leave not resulting in public reimbursement is not observed.

The Occupation and Industry Register from the Danish Occupational Cohort with eXposure data (DOC*X) (24) includes annual registrations of employment. Registrations of occupations are based on the Danish version of the International Standard Classification of Occupations from 1988 (DISCO-88). Industry is registered using the Danish extended 6-digit version (DB07) of the Nomenclature statistique des Activités économiques dans la Communauté Européenne (NACE). The register captures employment and occupation information from a citizen’s 16^th^ birthday or first registered employment until the last registered employment year.

*The Danish National Patient Registry* (25) captures both somatic and psychiatric diagnoses across inpatient and outpatient clinics from 1995 onwards. Furthermore, it comprises all discharge diagnoses from Danish somatic hospitals since 1977. The diagnoses are coded using the International Classification of Diseases, 8^th^ revision (ICD-8) between 1977 and 1993, and the ICD-10 classification system after 1993.

*The Danish National Prescription Registry* (26) documents every redeemed prescription from community pharmacies since 1995 and categorizes them in accordance with the anatomical therapeutic chemical (ATC) classification system.

*Administrative and demographic registers from Statistics Denmark* (19) capture a wide range of information such as income, education, sex, age, and death.

### Participants

Danish residents visiting an occupational medicine clinic in Denmark with a diagnosis of LBP (ICD-10 categories DM54.3, DM54.4 and DM54.5) were included if: (i) they were 18–60 years of age at the time of assessment, (ii) assessment took place between 2000 and 2017, (iii) they were not (voluntarily early) retired or on permanent health-related public benefits before the time of assessment and (v) they were living in Denmark at the time of assessment.

These criteria allowed for five years of labor market participation data before and after assessment.

For context, the patients can generally be viewed as patients with suspected work-related persistent LBP. In Denmark, referral to hospital departments of occupational medicine typically occurs when a general practitioner suspects a condition to be work-related (27). Patients referred with LBP will often have had symptoms for several months before occupational medicine consultation.

To contextualize findings, we selected five comparators from the general population for each patient, matched on birth year and sex. Comparators were randomly sampled with replacement from all individuals who fulfilled criteria (i), (ii) and (iii) on the patient’s index date. Each comparator was assigned the assessment date of the matched patient as their index date. Individuals from the comparison cohort who were later registered with a visit to an occupational medicine department for LBP were allowed to contribute to both cohorts to avoid informative censoring. The population size was determined by the number of unique patients seen during the study period.

### Labor market participation

Weekly labor market participation information from the DREAM register was used to group participants in 7 mutually exclusive categories: a) working, b) education or maternity/parental leave, c) temporary public benefits, d) permanent health-related public benefits, e) age-related public retirement or voluntary early retirement, f) emigration, and g) deceased. Education and parental leave were grouped because both reflect temporary statuses outside active work, with ongoing potential for later return to employment, unlike permanent exit states. Due to the eligibility criteria, permanent health-related public benefits, age-related retirement or voluntary early retirement, emigration, and death could not occur before the index date. Patients and comparators were separately grouped into these categories each week from five years before through five years after the index date. See supplementary material, www.sjweh.fi/article/4305, table S3 for specific codes used.

### Covariates

We expected patients with suspected work-related persistent LBP to differ socioeconomically and clinically from the general population (12, 18), so we accounted for these baseline differences to focus the description (28, 29) and isolate labor-market patterns attributable to the condition itself. The following covariates were used:

*Occupational information:* (i) D-ISCO 88 code providing information on job category on a 1-digit level eg, “managers”, “professionals” and “elementary occupations”. We collapsed the 10 original major D-ISCO 88 groups into six categories as shown in table 1 (27); (ii) Industrial classification DB07 (30) [Danish adaptation of NACE (31)] offering information on industry. We collapsed the original 21 sectors into nine categories as shown in table 1.

**Table 1:** Characteristics of patients with suspected work–related low-back pain (LBP) and comparators from the general population matched on age sex and index date.

Characteristic		General N=41 280		LBP N=8256
		N (%)		N (%)
Sex				
	Female	18 770 (45)		3754 (45)
	Male	22 510 (55)		4502 (55)
Age group (years)				
	<30	3710 (9.0)		742 (9.0)
	30–49	22 915 (56)		4583 (56)
	≥50	14 655 (36)		2931 (36)
Civil status				
	Unmarried	10 917 (26)		2208 (27)
	Married/partnership	24 222 (59)		4663 (56)
	Divorced/widowed	5028 (12)		1365 (17)
	Missing	1113 (2.7)		20 (0.2)
Educational level				
	Short	8844 (21)		3133 (38)
	Medium	18 990 (46)		4310 (52)
	Long	12 117 (29)		681 (8.2)
	Missing	1329 (3.2)		132 (1.6)
Average family income				
	Low	7809 (19)		1947 (24)
	Medium	23 663 (57)		5574 (68)
	High	9010 (22)		732 (8.9)
	Missing	798 (1.9)		3(<0.1)
Occupational classification				
	Armed forces and skill agricultural & fishery workers	826 (2.0)		166 (2.0)
	Clerks & service shop and market sales workers	10 346 (25)		2445 (30)
	Craft & related workers	4572 (11)		1299 (16)
	Elementary occupations	4747 (11)		2120 (26)
	Management legislation professionals technicians	15 756 (38)		734 (8.9)
	Plant & machine operators and assemblers	3242 (7.9)		1451 (18)
	Missing	1791 (4.3)		41 (0.5)
Collar				
	Blue-collar workers	13 387 (32)		5036 (61)
	White-collar workers	26 102 (63)		3179 (39)
	Missing	1791 (4.3)		41 (0.5)
Industry sector				
	Agriculture forestry & fishing	691 (1.7)		194 (2.3)
	Resource extraction manufacturing & supply	6463 (16)		1851 (22)
	Construction	2505 (6.1)		1018 (12)
	Trade repair transport & storage	7688 (19)		1378 (17)
	Information communication finance insurance real estate rent	2975 (7.2)		117 (1.4)
	Commercial services	3513 (8.5)		483 (5.9)
	Public administration education & health	12 464 (30)		2507 (30)
	Culture recreation & other services	1387 (3.4)		182 (2.2)
	Unknown	2406 (5.8)		513 (6.2)
	Missing	1188 (2.9)		13 (0.2)
Job change				
	No	29 279 (71)		6441 (78)
	Yes	10076 (24)		1765 (21)
	Unknown	1925 (4.7)		50 (0.6)
Labor status at index date				
	Working	34 095 (83)		3031 (37)
	Education or maternity/parental leave	1916 (4.6)		139 (1.7)
	Temporary public benefits	5269 (13)		5086 (62)
Somatic comorbidity (N)				
	0	29 844 (72)		4283 (52)
	1–2	10 373 (25)		3423 (41)
	>2	1063 (2.6)		550 (6.7)
Psychiatric comorbidity				
	No	39 521 (96)		7522 (91)
	Yes	1759 (4.3)		734 (8.9)
Index year				
	2000–2004	18 395 (45)		3679 (45)
	2005–2008	10 380 (25)		2076 (25)
	2009–2012	6460 (16)		1292 (16)
	2013–2017	6045 (15)		1209 (15)
Region of residency				
	Capital region	12 340 (30)		2259 (27)
	Zealand region	5882 (14)		1625 (20)
	Southern region	8582 (21)		1357 (16)
	Central region	9089 (22)		1454 (18)
	Northern region	4274 (10)		1541 (19)
	Missing	1113 (2.7)		20 (0.2)

*Sociodemographic information:* (i) Average five-year disposable household income up to one year before the index date (years -5– -1) grouped as low (first quintile), medium (second to fourth quintile) or high (last quintile) according to quintiles of the income distribution for each index year (32); (ii) Highest level of education attained before the index date. Based on the International Standard Classification of Education (ISCED), we used the following education groups: short (ISCED 1–3; primary, lower secondary and upper secondary), medium (ISCED 4; post-secondary non-tertiary education), and long (ISCED 5–8; short-cycle tertiary to doctoral level) (32); (iii) Age at index date, numeric in the propensity score (PS) models and categorized (whole years) in subgroup analyses: <30, 30–49, >49 years; (iv) Sex as registered at birth in the CPR register (male/female); (v) Period of occupational assessment/index, categorized: 2000–04, 2005–08, 2009–12, and 2013–17; (vi) Civil status (unmarried, married, divorced/widowed); (vii) Region of residency (Capital region, Zealand region, Southern region, Central region, Northern region).

*Comorbidity:* A comorbidity index used in previous Danish register-based studies (29, 33–35) was used to identify comorbidities based on discharge diagnoses from the Danish National Patient Register up to five years prior to the index date and filled prescriptions from the Danish National Prescription Register during the year up to the index date. For PS, we used disease counts within ten system categories: circulatory, endocrine, pulmonary, gastrointestinal, urogenital, musculoskeletal, hematological, and neurological systems, and cancers and mental health conditions. For definitions of subgroups in subsequent analyses, we used simpler categorizations (0, 1 or ≥2 somatic diseases and psychiatric disorders yes/no).

### Statistical analysis

The demographic characteristics of both the general and patient populations were described using counts and proportions. For both populations, we computed stacked area charts illustrating the weekly proportion of people in each of the seven mutually exclusive labor status groups during the five years before and after the index date (assessment date for patients) (18).

We used Poisson regression with cluster-robust standard errors (36, 37) to compute annual prevalence estimates with confidence intervals (CI) for years -5–5 (0=index date). In these analyses, prevalences were based on data from the weeks 52, 104, 156, 208, and 260 before and after the assessment/index date (eg, year 2 uses week 104 while year -2 uses week -104).

Inspired by Skajaa and colleagues (29), we performed a number of complementary analyses. Firstly, PS-weighted prevalence differences and ratios were computed for the above-mentioned time points comparing patients with the general population sample. The purpose of this analysis was to describe the differences in labor market participation between the groups while taking into account differences in measured covariates. PS were estimated using gradient boosting machines, which can improve covariate balance under weighting, reduce sensitivity to model misspecification, and flexibly accommodate non-linear relations and missing data when compared with traditional parametric models (38–40). Missing information on covariates were handled using missing indicators and non-linear relationships were handled in the gradient boosting algorithm.

In our case, the PS express the probability of being an LBP patient at a department of occupational medicine conditional on the covariates described above (41).

The patients were assigned weights of 1 while the comparators were assigned weights of PS/(1-PS), which corresponds to weighting for the average treatment effect on the treated. This approach reweights the comparator cohort so that its covariate distribution resembles that of the patient cohort (29).

Secondly, to detect effect measure modification, we recalculated the prevalence estimates as well as the PS-weighted prevalence differences and ratios, across subgroups at year 2. The subgroups were categorized by the covariate categories described above along with labor market status at index and job change defined as having a different D-ISCO code in year 2 post-index compared with both years 1 and 2 pre-index (people with unknown or missing D-ISCO codes were excluded in this subgroup analysis). For these analyses, the matching was not applied, and work status was coded positive (education, maternity/parental leave or working) or negative (all other statuses). PS weights were recalculated within each specific stratum examined. Individuals with missing information on the subgroup variable were excluded.

Thirdly, we conducted a supplementary analysis restricted to LBP patients receiving temporary public benefits (eg, sick leave) at the time of assessment/index focusing on return to work. In a time-to-event analysis, we used the Aalen-Johansen estimator with death, permanent health-related public benefits, and retirement as competing events to calculate the cumulative incidence of work resumption at six months and one, two, and five years after the assessment/index date. Emigration resulted in censoring. Work resumption was defined as three consecutive months (13 weeks) of no public benefits.

## Results

A total of 8256 patients with suspected work-related persistent LBP were included, along with 41 280 matched individuals from the general population. LBP patients had a markedly lower socioeconomic position at the index date: 38% had a short education (versus 21% in comparators), 61% worked in blue-collar occupations (versus 32%), only 9% held managerial or professional positions (versus 38%), and 26% worked in elementary occupations (versus 11%). They were overrepresented in industries such as construction (12% versus 6%) and resource extraction, manufacturing & supply (22% versus 16%) (table 1). Also, they were markedly more likely to suffer from somatic (48% versus 28%) and psychiatric (9% versus 4%) comorbidities. After PS-weighting, all measured covariates were well-balanced (maximum standardized mean difference=0.12). See supplementary figure S1 for PS distribution.

### Primary analysis

**Figure 1:**
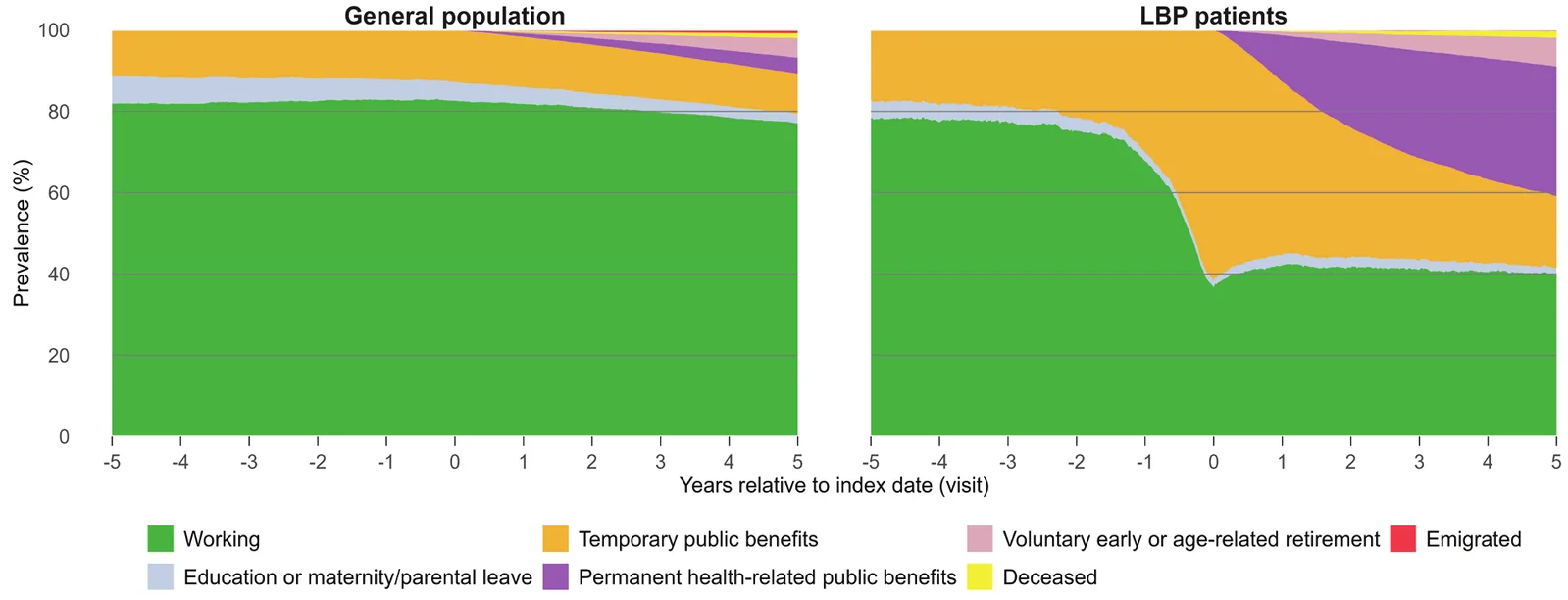
Stacked area chart of weekly prevalences of labor market states among occupational medicine low-back pain (LBP) patients and comparators from the general population matched on age, sex and index date.

Weekly labor market participation during five years before and after the index date is presented graphically for both cohorts in figure 1, while prevalence estimates of labor market status and PS-weighted prevalence differences (PSwPD) and ratios (PSwPR) are presented in table 2.

**Table 2:** Prevalence of labor market states relative to the index/assessment week among occupational medicine low-back pain (LBP) patients and comparators from the general population matched on age, sex and index date. [CI=confidence interval; PD=prevalence difference; PR=prevalence ratio; PS=propensity score]

Time ^a^	Status	General population		LBP patients		PS-weighted		PS-weighted
		% (95% CI)		% (95% CI)		PD (95% CI)		PR (95% CI)
-5 years	Working	82.0 (81.6–82.3)		78.3 (77.5–79.2)		0.9 (-0.4–2.2)		1.0 (1.0–1.0)
-5 years	Education or leave ^b^	6.7 (6.4–6.9)		4.3 (3.9–4.8)		-0.8 (-1.4– -0.2)		0.8 (0.7–1.0)
-5 years	Temporary public benefits	11.4 (11.1–11.7)		17.3 (16.5–18.2)		-0.1 (-1.3–1.1)		1.0 (0.9–1.1)
-4 years	Working	81.9 (81.6–82.3)		77.3 (76.4–78.2)		0.2 (-1.1–1.4)		1.0 (1.0–1.0)
-4 years	Education or leave ^b^	6.4 (6.1–6.6)		4.2 (3.8–4.6)		-0.8 (-1.4– -0.2)		0.8 (0.7–1.0)
-4 years	Temporary public benefits	11.7 (11.4–12.0)		18.6 (17.7–19.4)		0.7 (-0.5–1.9)		1.0 (1.0–1.1)
-3 years	Working	82.3 (81.9–82.6)		77.2 (76.3–78.1)		0.0 (-1.3–1.3)		1.0 (1.0–1.0)
-3 years	Education or leave ^b^	5.9 (5.7–6.2)		3.8 (3.4–4.3)		-0.7 (-1.2– -0.1)		0.9 (0.7–1.0)
-3 years	Temporary public benefits	11.8 (11.5–12.1)		18.9 (18.1–19.8)		0.6 (-0.6–1.8)		1.0 (1.0–1.1)
-2 years	Working	82.5 (82.2–82.9)		75.2 (74.3–76.1)		-1.0 (-2.4–0.3)		1.0 (1.0–1.0)
-2 years	Education or leave ^b^	5.5 (5.3–5.8)		3.3 (3.0–3.7)		-1.3 (-1.9– -0.7)		0.7 (0.6–0.8)
-2 years	Temporary public benefits	11.9 (11.6–12.3)		21.5 (20.6–22.4)		2.3 (1.0–3.6)		1.1 (1.1–1.2)
-1 year	Working	82.9 (82.5–83.3)		67.7 (66.7–68.7)		-8.3 (-9.7– -6.9)		0.9 (0.9–0.9)
-1 year	Education or leave ^b^	5.0 (4.8–5.2)		2.2 (1.9–2.5)		-2.1 (-2.6– -1.6)		0.5 (0.4–0.6)
-1 year	Temporary public benefits	12.1 (11.8–12.4)		30.1 (29.1–31.1)		10.4 (9.0–11.7)		1.5 (1.4–1.6)
Index	Working	82.6 (82.2–83.0)		36.7 (35.7–37.8)		-37.6 (-39.1– -36.2)		0.5 (0.5–0.5)
Index	Education or leave ^b^	4.6 (4.4–4.8)		1.7 (1.4–2.0)		-2.5 (-2.9– -2.0)		0.4 (0.3–0.5)
Index	Temporary public benefits	12.8 (12.4–13.1)		61.6 (60.6–62.7)		40.1 (38.7–41.5)		2.9 (2.7–3.0)
1 year	Working	81.9 (81.5–82.2)		42.1 (41.1–43.2)		-30.9 (-32.3– -29.4)		0.6 (0.6–0.6)
1 year	Education or leave ^b^	4.1 (3.9–4.3)		2.6 (2.3–3.0)		-1.2 (-1.7– -0.7)		0.7 (0.6–0.8)
1 year	Temporary public benefits	12.3 (12.0–12.6)		42.5 (41.5–43.6)		22.1 (20.6–23.5)		2.1 (2.0–2.2)
1 year	Retirement	0.4 (0.3–0.4)		0.9 (0.7–1.1)		0.4 (0.2–0.7)		1.8 (1.3–2.6)
1 year	Permanent health related public benefits	0.8 (0.8–0.9)		11.4 (10.8–12.1)		9.6 (8.8–10.4)		6.2 (5.0–7.7)
1 year	Emigrated	81.9 (81.5–82.2)		42.1 (41.1–43.2)		-30.9 (-32.3– -29.4)		0.6 (0.6–0.6)
1 year	Deceased	4.1 (3.9–4.3)		2.6 (2.3–3.0)		-1.2 (-1.7– -0.7)		0.7 (0.6–0.8)
2 years	Working	3.6 (3.4–3.7)		2.4 (2.1–2.8)		-1.0 (-1.5– -0.5)		0.7 (0.6–0.8)
2 years	Education or leave ^b^	11.9 (11.6–12.2)		31.9 (31.0–33.0)		12.8 (11.5–14.2)		1.7 (1.6–1.8)
2 years	Temporary public benefits	1.1 (1.0–1.2)		2.4 (2.1–2.7)		1.0 (0.6–1.3)		1.7 (1.4–2.1)
2 years	Retirement	1.6 (1.5–1.7)		20.9 (20.0–21.8)		17.5 (16.5–18.5)		6.1 (5.3–7.1)
2 years	Permanent health related public benefits	0.5 (0.4–0.5)		0.2 (0.1–0.4)		-0.1 (-0.2–0.0)		0.7 (0.4–1.2)
2 years	Emigrated	0.4 (0.3–0.5)		0.5 (0.4–0.7)		0.0 (-0.2–0.3)		1.1 (0.7–1.6)
2 years	Deceased	3.6 (3.4–3.7)		2.4 (2.1–2.8)		-1.0 (-1.5–(-0.5)		0.7 (0.6–0.8)
3 years	Working	79.7 (79.4–80.1)		41.1 (40.0–42.2)		-29.2 (-30.6– -27.7)		0.6 (0.6–0.6)
3 years	Education or leave ^b^	3.2 (3.0–3.3)		2.3 (2.0–2.6)		-0.8 (-1.2– -0.3)		0.7 (0.6–0.9)
3 years	Temporary public benefits	11.3 (11.0–11.6)		25.1 (24.2–26.1)		7.4 (6.1–8.7)		1.4 (1.3–1.5)
3 years	Retirement	2.2 (2.0–2.3)		3.9 (3.5–4.4)		1.3 (0.8–1.8)		1.5 (1.3–1.7)
3 years	Permanent health related public benefits	2.4 (2.2–2.5)		26.3 (25.4–27.3)		21.1 (20.0–22.3)		5.1 (4.5–5.7)
3 years	Emigrated	0.6 (0.5–0.7)		0.3 (0.2–0.5)		-0.1 (-0.2–0.1)		0.9 (0.6–1.3)
3 years	Deceased	0.6 (0.5–0.7)		0.9 (0.7–1.1)		0.1 (-0.1–0.4)		1.2 (0.9–1.6)
4 years	Working	78.5 (78.1–78.9)		40.4 (39.4–41.5)		-28.7 (-30.2– -27.2)		0.6 (0.6–0.6)
4 years	Education or leave ^b^	2.8 (2.6–3.0)		2.1 (1.8–2.5)		-0.7 (-1.2– -0.3)		0.7 (0.6–0.9)
4 years	Temporary public benefits	10.5 (10.2–10.8)		20.7 (19.8–21.6)		5.0 (3.8–6.2)		1.3 (1.2–1.4)
4 years	Retirement	3.4 (3.3–3.6)		5.4 (5.0–5.9)		1.4 (0.9–2.0)		1.4 (1.2–1.5)
4 years	Permanent health related public benefits	3.1 (3.0–3.3)		29.8 (28.9–30.8)		23.1 (21.9–24.3)		4.4 (4.0–4.9)
4 years	Emigrated	0.7 (0.6–0.8)		0.3 (0.2–0.4)		-0.2 (-0.4–(-0.1)		0.5 (0.3–0.9)
4 years	Deceased	0.9 (0.8–1.0)		1.3 (1.1–1.5)		0.1 (-0.2–0.4)		1.1 (0.8–1.4)
5 years	Working	77.1 (76.7–77.5)		39.8 (38.8–40.9)		-27.1 (-28.6– -25.6)		0.6 (0.6–0.6)
5 years	Education or leave ^b^	2.3 (2.2–2.4)		1.7 (1.4–2.0)		-0.7 (-1.1– -0.3)		0.7 (0.6–0.9)
5 years	Temporary public benefits	9.9 (9.6–10.2)		17.6 (16.8–18.5)		2.8 (1.7–4.0)		1.2 (1.1–1.3)
5 years	Retirement	4.9 (4.7–5.1)		7.1 (6.5–7.6)		1.6 (0.9–2.2)		1.3 (1.2–1.4)
5 years	Permanent health related public benefits	3.9 (3.7–4.1)		31.9 (30.9–32.9)		23.7 (22.4–24.9)		3.9 (3.5–4.3)
5 years	Emigrated	0.8 (0.7–0.9)		0.4 (0.2–0.5)		-0.3 (-0.5– -0.1)		0.6 (0.4–0.8)
5 years	Deceased	1.1 (1.0–1.2)		1.6 (1.3–1.9)		0.1 (-0.3–0.4)		1.0 (0.8–1.3)

^a^ Time relative to index date/date of assessment. Negative values indicate time before index date/date of assessment. The reported prevalences are point prevalences calculated for the specific week corresponding to each time point (eg, week 0=index; −2 years=week −104; +5 years=week +260). ^b^ Education or maternity/parental leave.

Five to two years before assessment, work prevalences for patients and comparators were roughly similar. A progressive decline was seen among patients at one year pre-index where work prevalence was 68% (versus 83% in comparators; PSwPD: -8.3%, PSwPR: 0.9) before declining to 37% at index (versus 83% in comparators; PSwPD:-37.6%, PSwPR:0.5).

Correspondingly, temporary public benefit prevalence among patients increased from 17% (versus 11% in comparators; PSwPD: -0.1%, PSwPR:1.0) at five years pre-index to 62% at index (versus 13% in comparators; PSwPD: 40.1%, PSwPR:2.9). Temporary public benefit prevalence among patients decreased from 43% at year one to 18% at year five, while permanent health-related public benefits prevalence increased from 11% to 32%. Work prevalence among LBP patients remained between 40–42% at all measured time points from one to five years post-index, approximately 40 percentage points below comparators (PSwPD -27% to -30% and PSwPR around 0.6). In the general population, work prevalence declined gradually over the ten-year period from 82% to 77%.

### Supplementary analyses

**Figure 2:**
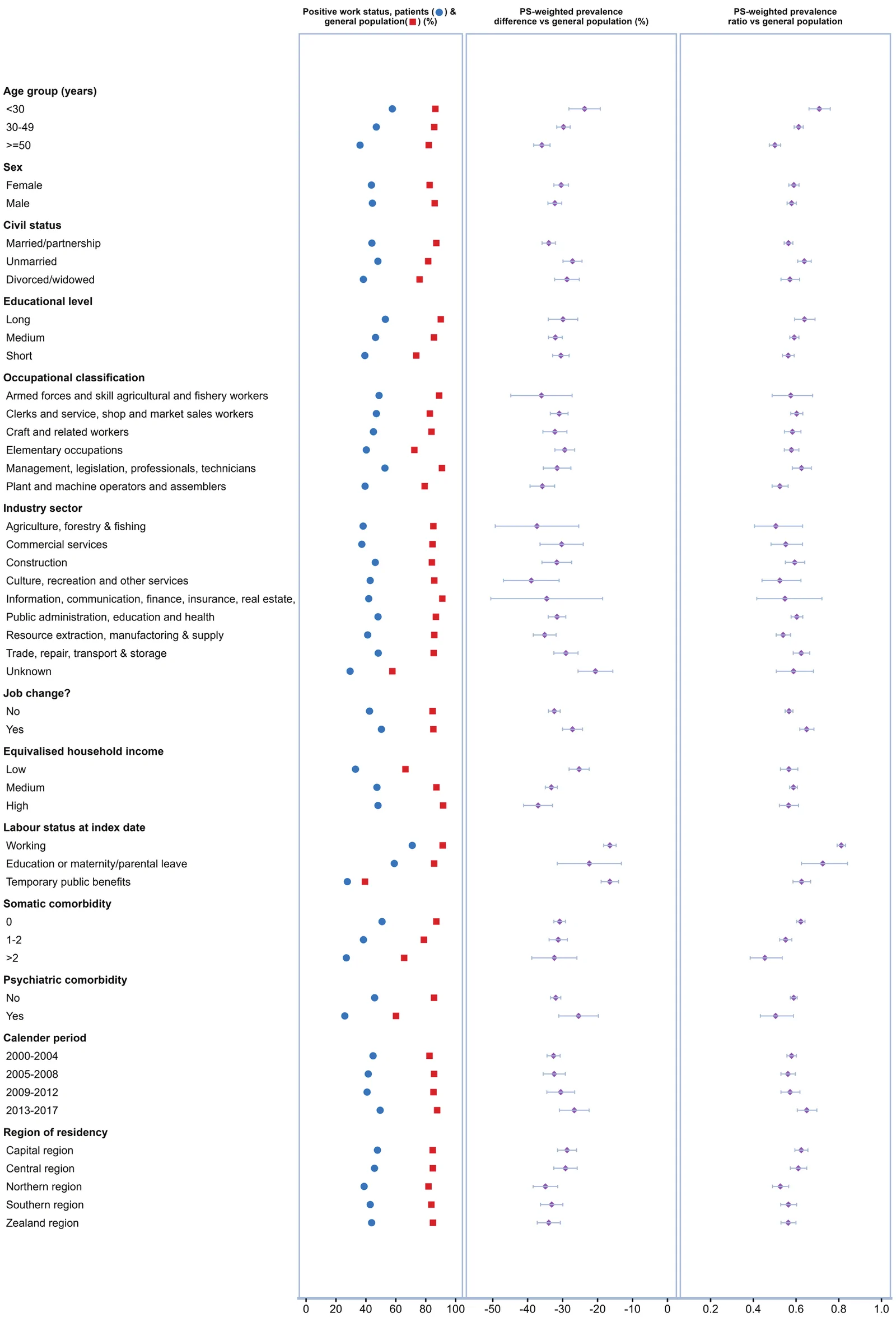
Prevalence of positive work status two years after assessment of low back pain at a department of occupational medicine (index date for the general population) with propensity-score-weighted differences and ratios comparing patients to the general population.

*Subgroup analyses.* Prevalence estimates of positive work status two years after index date are depicted in figure 2 and supplementary table S1. Patients with suspected work-related persistent LBP had consistently lower prevalences of positive work status across all characteristics, though the magnitude varied substantially across subgroups.

We observed a clear age gradient in working status; among younger patients (<30 years), 58% had a positive work status at two years with the smallest difference compared to the general population (PSwPD: -23.7%, PSwPR: 0.7), 47% had a positive work status among those aged 30–49 years (PSwPD: -29.8%, PSwPR: 0.6), while only 36% had a positive work status among those aged ≥50 years (PSwPD: -35.9%, PSwPR: 0.5). No meaningful differences were observed between men and women.

Labor market status at the index date was also strongly associated with work status at two years. However, PSwPD were largely similar while increasing contrasts were observed in PSwPR. Among patients working at index, 71% had a positive work status at two years compared with 91% in the general population (PSwPD: -16.5%; PSwPR: 0.8). Among those in education or on maternity/parental leave at index, the corresponding figures were 59% versus 86% (PSwPD: -22.4%; PSwPR: 0.7), and among those receiving temporary public benefits; 28% versus 39% (PSwPD: -16.5%; PSwPR: 0.6).

Socioeconomic indicators showed little-to-modest modification of work status, although absolute prevalences of positive work status were higher among patients with longer education and higher household income. Positive work status was 53% among patients with long education compared with 90% in comparators (PSwPD: -29.9%, PSwPR: 0.6), 47% versus 86% for those with medium-length education (PSwPD: -32.1%, PSwPR: 0.6), and 39% versus 74% for those with short education (PSwPD: -30.5%, PSwPR: 0.6). For household income strata, positive work status was 48% among patients with a high household income versus 92% in comparators (PSwPD: -37%, PSwPR: 0.6), 47% versus 87% (PSwPD: -33%, PSwPR: 0.6) among those with medium household income and 33% versus 66% (PSwPD: -25%, PSwPR: 0.6) among those with low household income.

A clear trend was observed for psychiatric and somatic comorbidities where absolute working prevalence was lower in groups defined by more comorbidity. However, groups with more comorbidities showed PSwPD and PSwPR that were lower or comparable to subgroups with less comorbidity. Positive work status at two years also differed by civil status. Among married or cohabiting patients, 44% had a positive work status compared with 87% in the general population (PSwPD: -34.0%; PSwPR: 0.6). The corresponding figures were 48% versus 82% among unmarried individuals (PSwPD: -27.2%; PSwPR: 0.6) and 38% versus 76% among those divorced or widowed (PSwPD: -28.8%; PSwPR: 0.6). Occupational and industry classifications revealed that manual labor and agricultural sectors were associated with the largest negative impacts. Positive work status at two years also varied by job change during follow-up. Among patients who did not change job, 42% had a positive work status compared with 85% in the general population (PSwPD: -32.4%; PSwPR: 0.6). Among those who changed job, the corresponding figures were 50% versus 85% (PSwPD: -27.2%; PSwPR: 0.7).

Note that balance in all covariates was not achieved in several subgroup analyses despite PS-weighting (supplementary table S1).

### Cumulative probability of returning to work

**Figure 3:**
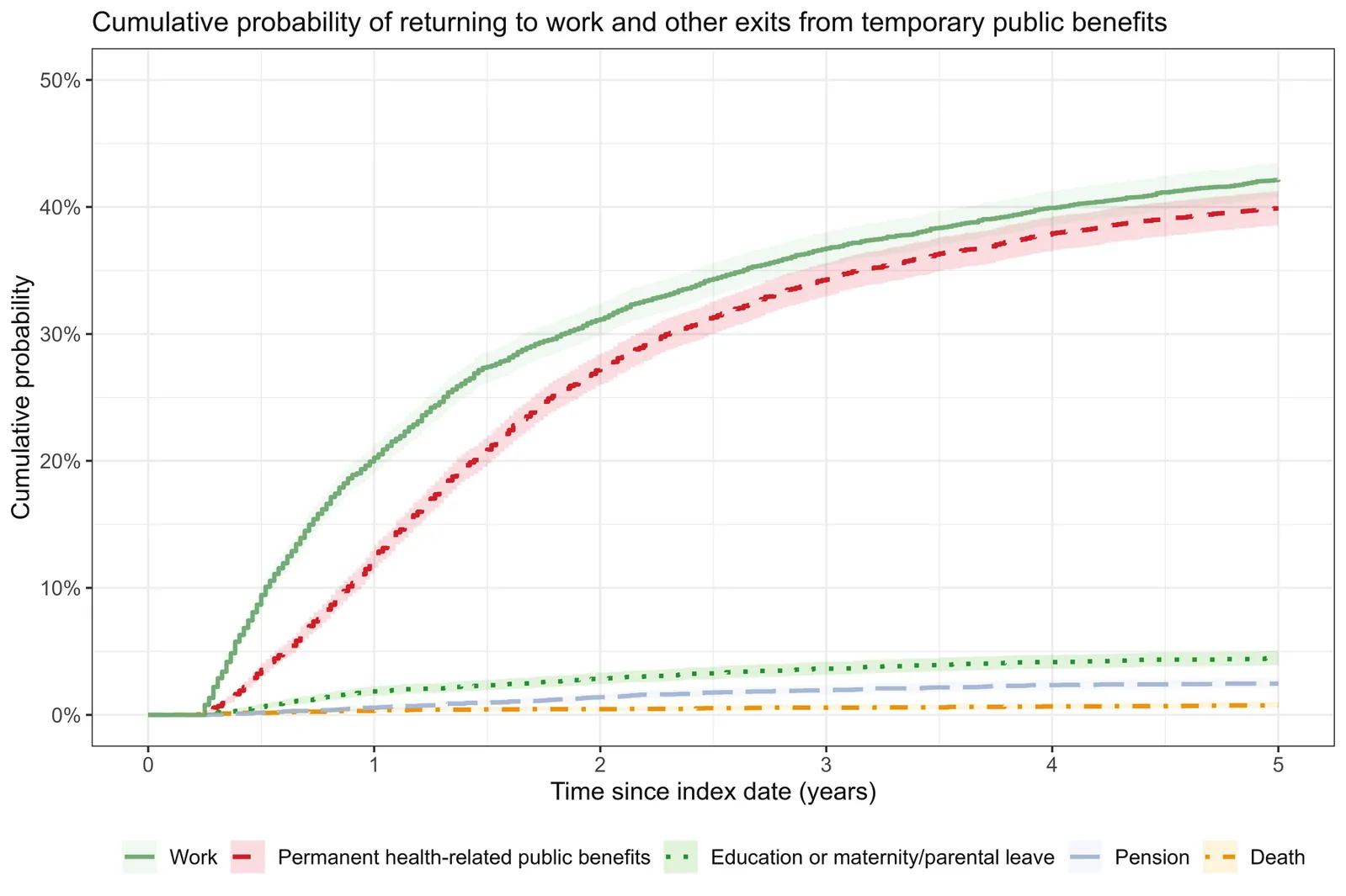
Cumulative probability of returning to work (work) and competing exits among occupational medicine low-back pain (LBP) patients.

Among the subset of LBP patients on temporary public benefits at the time of assessment (N=5086), the cumulative probability of returning to work was 9.4% (95% CI 8.7–10.3) at 6 months, 20.3% (95% CI 19.2–21.4) at 1 year, 31.2% (95% CI 29.9–32.4) at 2 years, and 42.1% (95% CI 40.8–43.5) at five years, accounting for death, retirement, and permanent health-related public benefits as competing events (figure 3 and supplementary table S2). Thus, the steepest increase occurred in the first two years, with more gradual increases thereafter, and less than half had returned after five years.

## Discussion

### Principal findings

Among patients with suspected work-related persistent LBP severe enough to require specialist occupational medicine evaluation, only 37% were working at assessment, compared to 83% of matched individuals from the general population. The majority of occupational LBP patients (62%) were receiving temporary public benefits at assessment/index, compared to only 13% in the general population.

Five years after assessment, employment remained substantially lower among LBP patients as compared to general population matches (40% versus 77%), representing a 37 percentage-point crude difference (PSwPD:-27.1%, PSwPR:0.6). Critically, about one-third of patients (32%) had transitioned to permanent health-related public benefits, eight times the rate among comparators (4%).

While the patient group and comparators had similar working prevalence at five years prior to assessment/index, patients showed declining labor market attachment at two years before assessment, with 22% receiving temporary public benefits compared to 12% in comparators. Previous studies have identified the first month of sick leave as a critical period for preventing long-term work absence, suggesting that Danish patients with LBP may be referred to occupational medicine departments at a relatively late stage, when opportunities for timely counselling and intervention may already be reduced (10).

While our competing risks analysis demonstrated that up to 42% of patients returned to work after temporary public benefits, the prevalence data reveal that the overall proportion employed increased only marginally from 37% at assessment to 40% after five years. This indicates that many patients who resume employment subsequently exit the workforce, suggesting that return to work following suspected work-related persistent LBP is often temporary rather than sustained. An alternative explanation could be that other individuals exit the work at a corresponding rate.

Compared with previous studies from occupational medicine settings, our findings suggest a less favorable work prognosis. In a Dutch study of workers consulting an occupational physician, around 90% had achieved occupational recovery within one year (17), whereas in our study only 42% were working one year after assessment. In the Dutch setting, workers typically consulted an occupational physician through an employer-contracted occupational health service after more than one week of sick leave or when considered at high risk of prolonged absence. In contrast, assessment in Danish hospital departments of occupational medicine usually requires referral from a general practitioner, and our data suggest that many patients had already been on sick leave for more than four weeks by the time of assessment. These differences in referral pathways, together with the likely longer symptom duration at consultation, may partly explain the poorer prognosis observed in our cohort. Otherwise, the observed tendencies are coherent with existing literature on the prognosis of persistent LBP. A meta-analysis (9) based on data from inception cohort studies found that prognosis of pain and disability was favorable in the first six weeks after study entry among patients with acute (<12 weeks) LBP, and to a lesser extent in patients with persistent (≥12 weeks) LBP. They concluded that patients presenting with persistent LBP could expect to still have moderate pain and disability after 12 months. In line with our subgroup analyses, factors such as higher age, worse health/comorbidities, poor outcome status at index date, employment in industries and occupations characterized by more manual labor and lower socioeconomic status seem to hamper the probability of a positive work status in LBP patients (8, 13, 15, 42, 43). However, our weighted contrast estimates were smaller among patients with more comorbidities and lower socioeconomic status. This pattern may reflect a floor effect: because labor market participation was already low in these groups, there was limited room for further reductions associated with suspected work-related persistent LBP. Not surprisingly, this mechanism was also apparent for poorer work status at the index date.

### Limitations, strengths and generalizability

To our knowledge this is the first large cohort study to examine labor market participation both before and after assessment at hospital departments of occupational medicine among patients with suspected work-related LBP, and to assess differences across potentially vulnerable subgroups. A major strength is the inclusion of *all* patients with LBP assessed on suspicion of work-relatedness at departments of occupational medicine in Denmark in 2000–2017, reducing the risk of selection bias.

Additionally, the utilization of detailed register data on demographics, health, and work-related variables minimized the risk of information being missing or wrongful. Further, we compared the labor market participation with that of the general population.

Some limitations of this study should be noted. Firstly, short-term sick leave (2–4 weeks) is not recorded in DREAM because these periods are paid by employers. As a consequence, individuals on short-term sick leave are classified as “working”, which leads to an overestimation of work participation in both cohorts. The magnitude of this overestimation depends on the proportion classified as working and may therefore differ slightly between patients and comparators. Around the time of the assessment, patients are likely to experience more short-term sick leave episodes that are not captured, but this differential misclassification is expected to diminish rapidly in the months following assessment. A general limitation of using public benefits to describe labor market participation is that public benefit categories, and thus work participation as defined by them, are sensitive to changes in policy and legislation. Major reforms of the Danish transfer-payment and benefit system relevant to labor market participation occurred during the study period, particularly in 2013–2014. These reforms may have influenced transitions across labor-market states and thereby affected both absolute prevalences and contrasts between patients and comparators. Although estimates were broadly similar across calendar periods, the somewhat smaller contrasts observed in 2013–2017 suggest that these reforms may partly have contributed to attenuated differences in the most recent period. Further, work participation is affected by the global and national economic landscape, with financial crises and similar events negatively affecting labor market participation.

A drawback of using register data is that we did not have information on individual factors such as lifestyle, pain coping behaviors, recovery expectations, symptom severity, subtype of LBP, clinical observations and tests, as well as workplace physical and psychosocial factors, which could all be important prognostic factors or modifiers for disability and return to work (13–15, 17, 42–44). Lastly, the study does not include all patients with suspected work-related persistent LBP but only those referred for assessment at a department of occupational medicine. These patients are mostly referred by their general practitioner or a medical specialist, and in some instances by their labor unions, on suspicion of work-relatedness. The population therefore most likely represents the most severe cases captured at a time when work capacity has already deteriorated and the LBP is persistent. The findings are likely to be most generalizable to patients assessed in specialist occupational medicine settings within tax-funded healthcare systems with universal access and similar social security arrangements, and more broadly to similar clinically selected populations with persistent LBP in whom work-relatedness is suspected. They are less likely to generalize directly to all patients with LBP, to workers seen earlier in the course of sickness absence, or to employer-based occupational health systems with easier access and different referral pathways.

### Concluding remarks

In this large cohort study of working-age individuals referred to occupational medicine departments with suspected work-related LBP, the prevalence of people working was consistently around 40% during the five years following assessment at a department of occupational medicine. Labor market participation worsened from two years prior to assessment, indicating a possible need for earlier referral. Prognosis was particularly poor among older patients, those with weaker labor market participation at assessment, and those with greater comorbidity. Of individual patients on temporary public benefits at the time of assessment, about 42% returned to work during the following five years. However, return to work was likely temporary rather than sustained. These findings underscore the need for further research into earlier identification, intervention, and specialist referral.

## Supplementary material

Supplementary material

## Data Availability

The data can be acquired through Statistics Denmark under their rules and regulations: Data for research–Statistics Denmark. If you wish to acquire data identical to the data used in this study once you have the necessary permissions from Statistics Denmark, feel free to contact the corresponding author for data specifications.
