# Supplementary material for: Labor market participation among patients referred to occupational medicine with low-back pain: a Danish nationwide register-based cohort study
Source: Scand J Work Environ Health. 2026 Aug 31;52(5):593–605. doi: 10.5271/sjweh.4305 (PMC13554461; doi:10.5271/sjweh.4305)
Supplement: Supplementary file 1 [file SJWEH-52-593-S001.pdf]

# Labor market participation among patients referred to occupational medicine with low-back pain: a Danish nationwide register-based cohort study[1]

*by Mathias Moselund Rønnow, MSc,* [2] *Jesper Medom Vestergaard, PhD, Dorte Rytter, PhD, Morten Fenger-Grøn, PhD, Morten Vejs Willert, PhD, Johan Hviid Andersen, PhD, Marianne Kyndi, PhD*[2]

1. Supplementary material
2. Correspondence to: Mathias Moselund Rønnow, Department of Occupational and Environmental Medicine–University Research Clinic, Danish Ramazzini Center, Goedstrup Regional Hospital, Herning, Denmark. [E-mail: mathro@rm.dk] ORCID: https://orcid.org/0000-0003-0678-0901

**Labor market participation among patients referred to occupational medicine with low-back pain: a Danish nationwide register-based cohort study**

**Figure S1:** Propensity score distribution before and after weighting in the subsample of the general working population (general) and lower back pain patients (LBP).

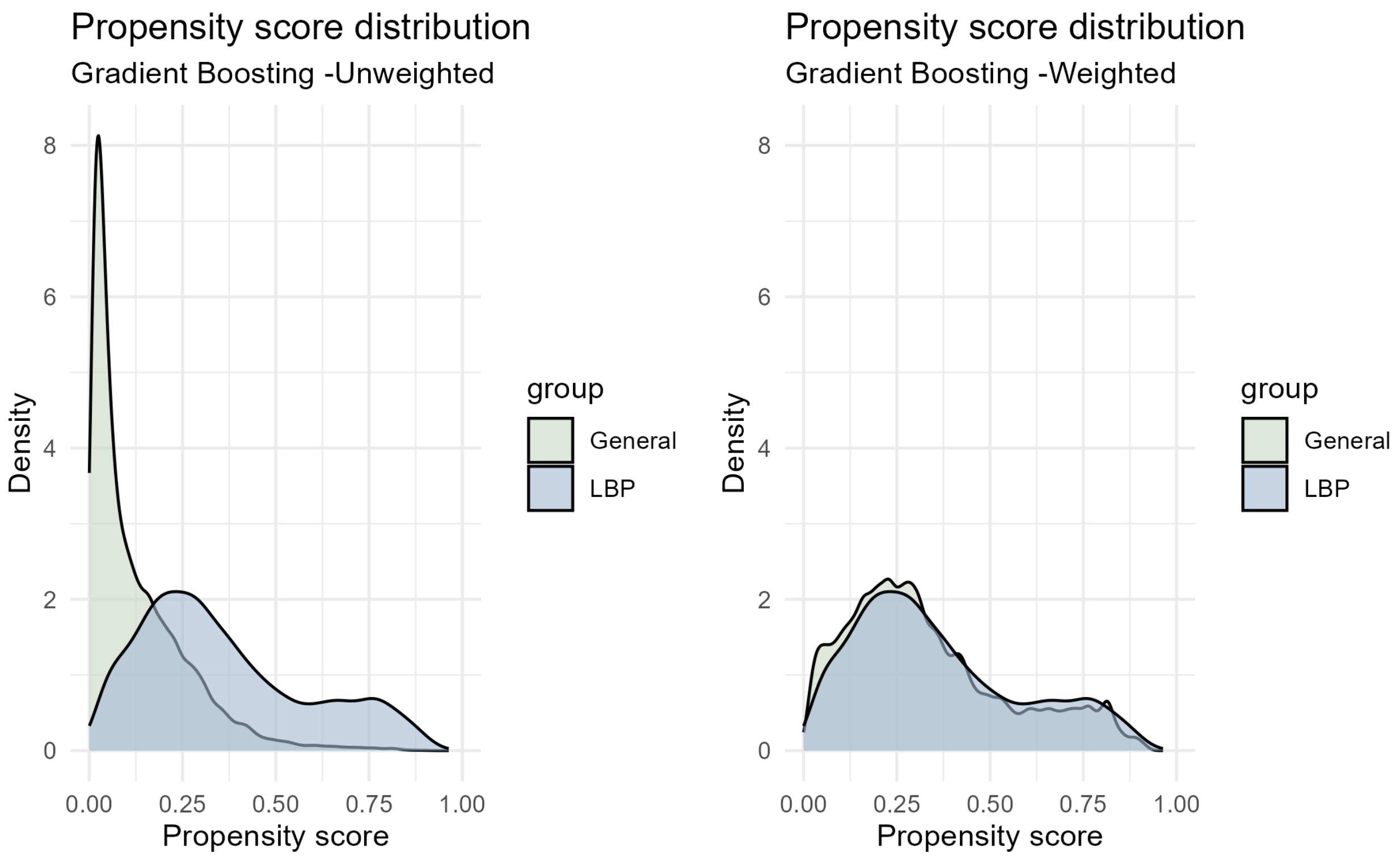


We used gradient boosting with stopping criteria “es.max” to estimate propensity score weights using the average treatment effect in the treated (ATT). Missing values were handled using missing-indicator coding, where each variable with missingness was assigned a separate indicator and missing values were replaced with arbitrary constants for model fitting (”ind” option in the WeighIt package). The WeightIt package was used (Greifer N (2025). *WeightIt: Weighting for Covariate Balance in Observational Studies*. R package version 1.5.0, https://ngreifer.github.io/WeightIt/.)

| **Table S1. Subgroup analyses of prevalences (prev.) of positive labor market status at 2 years after index*** | | | | | | | | |
|---|---|---|---|---|---|---|---|---|
| Subgroup (index time values) | LBP[a] working prev., % | General pop.[b] working prev., % | PS[c]-weighted difference (95% CI[d]) vs general | PS[c]-weighted ratio (95% CI[d]) vs general | LBP[a] patients, n | General pop.[b], n | General pop.[b], ESS[e] | Max SMD[f] |
| Age group (years) | | | | | | | | |
| <30 | 57.7 | 86.4 | -23.71 (-28.21 – -19.22 | 0.71 (0.66 – 0.76) | 742 | 3710 | 773 | 0.29 |
| 30-49 | 47.0 | 85.7 | -29.75 (-31.66 – -27.83 | 0.61 (0.59 – 0.63) | 4583 | 22915 | 5619 | 0.12 |
| >=50 | 36.1 | 82.0 | -35.93 (-38.27 – -33.59 | 0.50 (0.48 – 0.53) | 2931 | 14655 | 3799 | 0.17 |
| Sex | | | | | | | | |
| Male | 44.3 | 86.0 | -32.21 (-34.20 – -30.23 | 0.58 (0.56 – 0.60) | 4502 | 22510 | 4596 | 0.17 |
| Female | 43.7 | 82.6 | -30.42 (-32.51 – -28.32 | 0.59 (0.57 – 0.61) | 3754 | 18770 | 5058 | 0.13 |
| Civil status | | | | | | | | |
| Married/partnership | 44.0 | 87.1 | -33.96 (-35.88 – -32.03 | 0.56 (0.54 – 0.59) | 4663 | 24222 | 5562 | 0.10 |
| Unmarried | 48.0 | 81.6 | -27.16 (-29.89 – -24.43 | 0.64 (0.61 – 0.67) | 2208 | 10917 | 2586 | 0.20 |
| Divorced/widowed | 38.3 | 75.9 | -28.75 (-32.28 – -25.22 | 0.57 (0.53 – 0.62) | 1365 | 5028 | 1698 | 0.26 |
| Educational level | | | | | | | | |
| Short | 39.3 | 73.7 | -30.49 (-32.83 – -28.14 | 0.56 (0.54 – 0.59) | 3133 | 8844 | 3560 | 0.20 |
| Medium | 46.5 | 85.5 | -32.08 (-34.05 – -30.10 | 0.59 (0.57 – 0.61) | 4310 | 18990 | 5435 | 0.11 |
| Long | 53.0 | 90.1 | -29.89 (-34.10 – -25.68 | 0.64 (0.59 – 0.69) | 681 | 12117 | 1675 | 0.34 |
| Occupational classification | | | | | | | | |
| Plant and machine operators and assemblers | 39.4 | 79.3 | -35.79 (-39.31 – -32.28 | 0.52 (0.49 – 0.56) | 1451 | 3242 | 1415 | 0.37 |
| Management, legislation, professionals, technicians | 52.7 | 90.8 | -31.58 (-35.54 – -27.62 | 0.63 (0.58 – 0.67) | 734 | 15756 | 2906 | 0.30 |
| Elementary occupations | 40.3 | 72.4 | -29.39 (-32.22 – -26.55 | 0.58 (0.55 – 0.61) | 2120 | 4747 | 2338 | 0.27 |
| Craft and related workers | 45.0 | 83.9 | -32.20 (-35.63 – -28.76 | 0.58 (0.55 – 0.62) | 1299 | 4572 | 2001 | 0.17 |
| Clerks and service, shop and market sales workers | 47.0 | 82.8 | -30.96 (-33.50 – -28.43 | 0.60 (0.58 – 0.63) | 2445 | 10346 | 3304 | 0.14 |
| Armed forces and skill agricultural and fishery workers | 48.8 | 89.0 | -36.05 (-44.83 – -27.28 | 0.58 (0.49 – 0.68) | 166 | 826 | 299 | 0.74 |

| | | | | | | | | |
|---|---|---|---|---|---|---|---|---|
| Industry sector | | | | | | | | |
| Unknown | 29.4 | 57.7 | -20.61 (-25.59 – -15.63 | 0.59 (0.51 – 0.68) | 513 | 2406 | 1039 | 0.29 |
| Trade, repair, transport & storage | 48.3 | 85.4 | -29.05 (-32.51 – -25.59 | 0.62 (0.59 – 0.66) | 1378 | 7688 | 1922 | 0.17 |
| Resource extraction, manufactoring & supply | 41.2 | 85.7 | -35.14 (-38.41 – -31.87 | 0.54 (0.51 – 0.57) | 1851 | 6463 | 1751 | 0.26 |
| Public administration, education and health | 48.1 | 86.8 | -31.60 (-34.12 – -29.08 | 0.60 (0.58 – 0.63) | 2507 | 12464 | 2960 | 0.12 |
| Information, communication, finance, insurance, real estate, rent | 41.9 | 91.1 | -34.55 (-50.54 – -18.56 | 0.55 (0.42 – 0.72) | 117 | 2975 | 106 | 0.80 |
| Culture, recreation and other services | 42.9 | 85.7 | -38.96 (-46.92 – -30.99 | 0.52 (0.44 – 0.62) | 182 | 1387 | 585 | 0.45 |
| Construction | 46.3 | 84.1 | -31.66 (-35.91 – -27.42 | 0.59 (0.55 – 0.64) | 1018 | 2505 | 960 | 0.27 |
| Commercial services | 37.3 | 84.6 | -30.28 (-36.45 – -24.10 | 0.55 (0.48 – 0.63) | 483 | 3513 | 597 | 0.45 |
| Agriculture, forestry & fishing | 38.1 | 85.1 | -37.34 (-49.30 – -25.38 | 0.51 (0.40 – 0.63) | 194 | 691 | 145 | 1.14 |
| Job change? | | | | | | | | |
| No | 42.4 | 84.6 | -32.38 (-34.06 – -30.71 | 0.57 (0.55 – 0.59) | 6441 | 29279 | 7130 | 0.11 |
| Yes | 50.4 | 85.1 | -27.17 (-30.03 – -24.30 | 0.65 (0.62 – 0.68) | 1765 | 10076 | 2639 | 0.21 |
| Equivalised household income | | | | | | | | |
| Low | 33.0 | 66.4 | -25.28 (-28.15 – -22.42 | 0.57 (0.53 – 0.61) | 1947 | 7809 | 2552 | 0.19 |
| Medium | 47.4 | 87.2 | -33.22 (-34.93 – -31.52 | 0.59 (0.57 – 0.61) | 5574 | 23663 | 6479 | 0.11 |
| High | 48.1 | 91.6 | -37.01 (-41.14 – -32.88 | 0.57 (0.52 – 0.61) | 732 | 9010 | 1393 | 0.37 |
| Labor market status at index date | | | | | | | | |
| Working | 71.0 | 91.4 | -16.47 (-18.25 – -14.69 | 0.81 (0.79 – 0.83) | 3031 | 34095 | 9599 | 0.21 |
| Education or maternity/parental leave | 59.0 | 85.6 | -22.36 (-31.55 – -13.16 | 0.73 (0.63 – 0.84) | 139 | 1916 | 361 | 1.32 |
| Temporary public benefits | 27.6 | 39.3 | -16.50 (-18.97 – -14.02 | 0.63 (0.59 – 0.67) | 5086 | 5269 | 2067 | 0.15 |
| Somatic comorbidity | | | | | | | | |
| >2 | 26.9 | 65.6 | -32.36 (-38.82 – -25.90 | 0.45 (0.39 – 0.53) | 550 | 1063 | 310 | 0.45 |
| 1-2 | 38.4 | 78.8 | -31.26 (-33.87 – -28.64 | 0.55 (0.52 – 0.58) | 3423 | 10373 | 2219 | 0.24 |

| | | | | | | | | |
|---|---|---|---|---|---|---|---|---|
| 0 | 50.8 | 87.1 | -30.84 (-32.52 – -29.16 | 0.62 (0.60 – 0.64) | 4283 | 29844 | 11398 | 0.15 |
| Psychiatric comorbidity | | | | | | | | |
| Yes | 25.9 | 60.1 | -25.44 (-31.10 – -19.77 | 0.50 (0.43 – 0.59) | 734 | 1759 | 440 | 0.60 |
| No | 45.8 | 85.6 | -31.97 (-33.42 – -30.51 | 0.59 (0.57 – 0.61) | 7522 | 39521 | 9560 | 0.10 |
| Calendar period | | | | | | | | |
| 2013-2017 | 49.5 | 87.6 | -26.66 (-30.90 – -22.42 | 0.65 (0.61 – 0.70) | 1209 | 6045 | 852 | 0.27 |
| 2009-2012 | 40.8 | 85.2 | -30.53 (-34.51 – -26.55 | 0.57 (0.53 – 0.62) | 1292 | 6460 | 1154 | 0.25 |
| 2005-2008 | 41.6 | 85.6 | -32.40 (-35.57 – -29.24 | 0.56 (0.53 – 0.60) | 2076 | 10380 | 1787 | 0.15 |
| 2000-2004 | 44.8 | 82.5 | -32.60 (-34.47 – -30.73 | 0.58 (0.56 – 0.60) | 3679 | 18395 | 7885 | 0.20 |
| Region of residency | | | | | | | | |
| Zealand region | 43.8 | 84.8 | -33.96 (-37.27 – -30.65 | 0.56 (0.53 – 0.60) | 1625 | 5882 | 1563 | 0.32 |
| Southern region | 42.9 | 83.8 | -33.12 (-36.31 – -29.93 | 0.56 (0.53 – 0.60) | 1357 | 8582 | 2773 | 0.21 |
| Northern region | 38.8 | 81.9 | -34.92 (-38.44 – -31.39 | 0.53 (0.49 – 0.57) | 1541 | 4274 | 1334 | 0.28 |
| Central region | 45.7 | 84.8 | -29.18 (-32.54 – -25.82 | 0.61 (0.57 – 0.65) | 1454 | 9089 | 2216 | 0.18 |
| Capital region | 47.7 | 84.7 | -28.72 (-31.44 – -26.00 | 0.62 (0.59 – 0.66) | 2259 | 12340 | 2639 | 0.24 |

*All comparisons are weighted using propensity scores (ATT). Differences and ratios compare LBP patients to the general population

[a] LBP= Patients with suspected work-related persistent low back pain

[b] General pop.= A sub-sample of the general working population

[c] PS= Propensity score

[d] CI= Confidence interval (95%)

[e] Effective sample size in general population used for PS-weighted differences and ratios (after general population has been down-weighed)

[f] Max SMD= The maximum standardized mean difference per strata after weighting that was observed among covariates.

Figure 2 in the main manuscript is based on the data presented in this table.

**Table S2:** Cumulative probability of returning to work (defined as 3 months without public benefits) among patients receiving temporary public benefits at time of assessment (n=5,086). The table includes competing exits from temporary public benefits.

| Exit reason | 6 months | 1 years | 1.5 years | 2 years | 3 years | 4 years | 5 years |
|---|---|---|---|---|---|---|---|
| Work | 9.44% (8.66%–10.26%) | 20.26% (19.16%–21.37%) | 27.42% (26.20%–28.65%) | 31.16% (29.90%–32.44%) | 36.74% (35.42%–38.07%) | 39.93% (38.58%–41.27%) | 42.14% (40.78%–43.49%) |
| Pension | 0.20% (0.10%–0.35%) | 0.57% (0.39%–0.81%) | 0.96% (0.72%–1.26%) | 1.40% (1.10%–1.75%) | 1.95% (1.60%–2.36%) | 2.34% (1.95%–2.79%) | 2.46% (2.06%–2.92%) |
| Permanent health-related public benefits | 3.54% (3.06%–4.07%) | 12.45% (11.56%–13.37%) | 20.89% (19.79%–22.02%) | 27.26% (26.04%–28.48%) | 34.30% (32.99%–35.60%) | 37.93% (36.60%–39.26%) | 39.89% (38.54%–41.23%) |
| Education or maternity/parental leave | 0.63% (0.44%–0.88%) | 1.85% (1.51%–2.25%) | 2.34% (1.95%–2.78%) | 2.85% (2.42%–3.34%) | 3.64% (3.15%–4.18%) | 4.16% (3.63%–4.73%) | 4.47% (3.93%–5.06%) |
| Death | 0.16% (0.08%–0.30%) | 0.33% (0.20%–0.53%) | 0.43% (0.28%–0.65%) | 0.43% (0.28%–0.65%) | 0.57% (0.39%–0.81%) | 0.67% (0.47%–0.93%) | 0.75% (0.54%–1.02%) |

**Table S3: DREAM codes used for classification:**

| Status | Codes |
|---|---|
| Working | No code, 121, 122, 123, 996 |
| Education or maternity/parental leave | 151, 152, 153, 412, 413, 521, 651, 652, 661, 881 |
| Temporary public benefits | 124, 125, 126, 111, 112, 123, 115, 130, 131, 132, 133, 134, 135, 136, 137, 138, 139, 140, 143, 144, 145, 146, 147, 148, 149, 160, 163, 164, 165, 166, 167, 168, 169, 211, 121, 213, 214, 215, 216, 217, 218, 219, 231, 232, 299, 511, 522, 611, 700, 703, 704, 705, 706, 707, 708, 709, 710, 713, 714, 715, 716, 717, 718, 719, 720, 723, 724, 725, 726, 727, 728, 729, 730, 733, 734, 735, 736, 737, 738, 739, 750, 752, 753, 754, 755, 756, 757, 758, 760, 762, 763, 764, 765, 766, 767, 768, 810, 813, 814, 815, 816, 817, 818, 870, 873, 874, 875, 876, 877, 878, 890, 892, 893, 894, 895, 896, 897, 898, 899 |
| Permanent health-related public benefits | 740, 741, 742, 743, 744, 745, 746, 747, 748, 771, 772, 773, 774, 781, 782, 783, 784, 785 |
| Voluntary early or age-related retirement | 621, 622, 786, 998 |
| Death | 999 |
| Emigrated | 997 |
